# The Effects of Emulsifier Addition on the Functionalization of a High-Oleic Palm Oil-Based Oleogel

**DOI:** 10.3390/gels9070522

**Published:** 2023-06-27

**Authors:** Melissa Perez-Santana, Victor Cedeno-Sanchez, John C. Carriglio, Andrew J. MacIntosh

**Affiliations:** Food Science and Human Nutrition Department, University of Florida, Gainesville, FL 32611, USA

**Keywords:** high-oleic palm oil, oleogel, shortening, hardness, DSC

## Abstract

Alternatives to oils with high saturated fatty acid content are often liquid oils (high in unsaturated fatty acids) that have a modified structure created either through additives or processing. Emulsifiers are additives that can be used as structuring agents of liquid fats; this process results in products such as oleogels, which can broaden the applications of these oils. This study assessed and compared the effects of mono- and diglycerides at 3%, 5%, 7% and 10% *w*/*w* on the mechanical and thermal properties of high-oleic palm oil (HOPO) oleogels. HOPO was heated to 75 °C and mixed with mono- or diglycerides at those four concentrations. The thermomechanical properties of the melted oleogels were assessed using differential scanning calorimetry (DSC). The melted oleogels were cooled to final temperatures of 5 °C, 10 °C and 15 °C under identical cooling rates, after which a puncture test (via a texture analyzer) was used to assess their textures. Finally, polarized light microscopy was used to assess the mechanical changes induced through emulsifier addition. The results showed that the use of mono- and diglycerides significantly modified the thermal and mechanical properties of the oleogels. The addition of saturated monoglycerides promoted a higher-temperature nucleation stage that did not previously occur in HOPO. The onset crystallization temperature increased with the addition of diglycerides, promoting crystallization at higher temperatures of the high-melting fraction of HOPO. The hardness of the oleogel generally increased with emulsifier addition and a reduction of the temperature. The effect of the temperature on the hardness was significantly greater in the diglyceride oleogel than in the monoglyceride oleogel. This study shows that the addition of mono- and diglycerides allows companies to customize their formulations to achieve desired results that may not previously have been possible, thereby facilitating novel uses for these oils within the industry.

## 1. Introduction

The American Heart Association recommends that vegetable oils and other fats comprise 20–35% of daily calorie consumption [1]. In 2015, a ban on partially hydrogenated oils in food products produced in or imported to the United States led to the introduction of oils that were high in saturated fatty acids (SFAs) being used as alternatives. Under proper processing conditions, oils that are high in SFAs have similar mechanical properties to partially hydrogenated oils in food applications [2]. However, negative health effects have also been associated with SFAs [3,4]. Therefore, the consumption of oils rich in mono- and polyunsaturated fatty acids is recommended by major health institutions. These present a challenge for food developers, as these oils are typically liquid at room temperature or have poor mechanical performance. Techniques have been developed to create a stable fat matrix at room temperature to broaden the applications of more nutritious liquid oils. One method to achieve this uses low-molecular-weight structuring agents (emulsifiers) and crystallization techniques that promote liquid oil entrapment. Some of these configurations are referred to as oleogels and have been increasingly studied over the last twenty years [5,6,7] to replace specific fats that are high in SFAs. However, industrial use remains limited, partially due to structuring agents being specific for particular applications and the need for more detailed studies on the shelf-life, sensory perception, GRAS status and effects on health, among other factors, for each oleogelator [8,9]. The objective of this study was to analyze the effects of fat-based emulsifiers, mono- and diglycerides, on the properties of oleogels made with a novel palm oil that is low in SFAs.

Common oils that are low in SFAs have been studied for potential oleogel applications, including health-contributing high-oleic varieties of soybean and canola [10,11]. Standard palm oil fractionated to a lower saturated fat content and mixed with beeswax as oleogelator has also been studied, producing soft oleogels with similar melting points compared to commercial shortenings [12]. However, palm oil is one of the most widely used oils around the world, and there is extremely limited information on the use of palm oil with a higher content of oleic acid in oleogels. High-oleic palm oil (HOPO) is extracted from the hybrid palm *E. guineensis* and *E. oleifera* OxG; this palm has slower growth and is more resistant to bud rot disease, which increases its productive life and reduces the need for cut down and replanting, potentially decreasing deforestation rates [13,14]. HOPO has a higher concentration of monounsaturated oleic acid, with 53.5–55.3% of the total fatty acids (FAs), than regular palm oil from *E. guineensis* (37% total FAs), resulting in a lower concentration of saturated fatty acids in HOPO (30%) without the need for fractionation. This oil is soft and semisolid at room temperature and contains high concentrations of bioactive compounds, such as vitamin A carotenes and Vitamin E α-tocopherol [15]. HOPO has approximately 2.5% *w*/*w* naturally occurring DAGs, which slow nucleation in regular palm oil, and a low concentration of fully saturated triglycerides (1.5%) [16]. Commercial applications of HOPO could be broadened by additional gelling agents or emulsifiers that form oleogels [11].

Availability, cost, health benefits and/or gelling properties are important considerations for structuring agents. The molecular structures of monoglycerides (MAGs) make them appealing for use as gelling agents [7]. The hydrophilic heads of MAGs are stabilized by hydrogen bonds, while the affinity of the hydrophobic tail with the triglycerides stabilizes the structure and promotes crystallization [17]. A similar principle applies to diglycerides (DAGs), which improve oil retention because of an additional aliphatic chain in the glycerol backbone. An incentive to use oleogels compared to pure oils relates to human health-promoting effects. In vivo and in vitro studies on have shown that oleogels delay lipid absorption by reducing the lipolysis rate [18]. An application of this effect has shown that the addition of lipid-soluble bioactive compounds such as B-carotene can extend its release times, while the bioavailability of curcumin is increased [8]. DAGs have also shown low absorption rates in animal models [19] and slower in vitro lipolysis when used above a certain threshold [20]. As carriers of bioactive compounds, 20% MAG oleogels exhibited positive correlations with B-carotene stability, keeping up to 72% of the bioactive compound after heat or UV exposures [21].

The processing conditions in oleogel manufacturing are as important to the stability of the oleogel as the ingredients. Critical processing parameters include shearing, crystallization temperature and conditioning. Additionally, supplemental processes, such as high-intensity ultrasound (HIU)-induced crystallization, have been shown to increase oil retention in oleogels [6]. Da Pieve et al. [5] observed that shear force applied during crystallization weakens cod liver oleogels stabilized with monoglycerides, mainly due to the rupturing of the gel-like structure. Giacomozzi et al. [6] observed that higher cooling rates could be used to stabilize a high-oleic sunflower oil with monoglycerides, identifying that higher cooling rates and higher monoglyceride content improve the viscoelastic and thermal properties of the oil. The addition of DAGs has been studied to replace different commercial fats, and using sunflower oil at different shearing and cooling rates [8], in-depth characterization has shown that oleogels with DAG mixtures can result in structures with different brittleness under shear depending on the type of stirrer [8].

The use of the herein proposed MAG and DAG oleogels has a widespread range found in the literature [22]. Applications in products such as chocolate, using oleogels with 10% MAGs, were able to replace the 100% cocoa butter in dark chocolate formulation [23]. In praline model-systems, MAGs used at 3% were able to replace confectionery filing fats [24]. In meat products, oleogels containing 15% MAGs, 5% phytosterols were used to replace animal fats in pork frankfurter sausages [25], with 15% MAGs in fermented sausages [26] and 5% MAGs for bologna sausages [27] replacing up to 50% of the pork fat in the three studies. As a replacement for bakery fats, oleogels containing 6–8% MAGs have been able to generate uniform volume and crumb size while improving oil and water retention in products such as muffins, reducing 80% of the saturated fat [28]. For oleogels to replace existing partially hydrogenated and oils that are high in SFAs, the physical characteristics must be comparable. Differential scanning calorimetry (DSC) analysis can be used to identify the optimal concentration of structuring agent that modifies the crystallization temperature and melting point. Puncture testing is a fast and reliable technique to assess changes in the hardness and spreadability of oleogels. Other techniques, such as oscillatory rheology, oil-binding capacity, and microscopy, are also tools that describe the performance and characteristics of oleogels at different operational conditions.

To the knowledge of the authors, changes in the behavior of HOPO caused by the addition of emulsifiers have not been studied. The specific objectives of this study were to assess and compare the effects of added mono- and diglycerides on the mechanical and thermal properties of HOPO oleogels. This assessment was conducted through the use of DSC, texture analysis and microscopy tests.

## 2. Results and Discussion

### 2.1. Differential Scanning Calorimetry for MAG and DAG Oleogels

**Figure 1:**
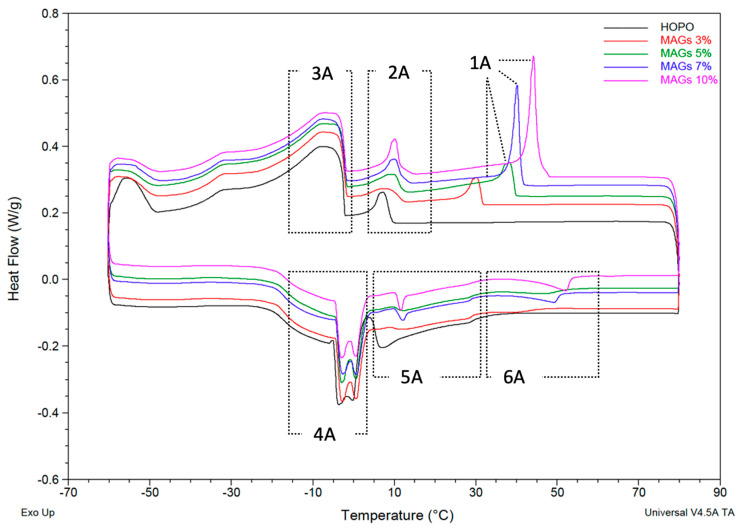
Thermogram for HOPO (black) and MAGs at 3%, 5%, 7% and 10% *n* = 2, with each baseline heat flow offset from HOPO (black) for clarity.

**Figure 2:**
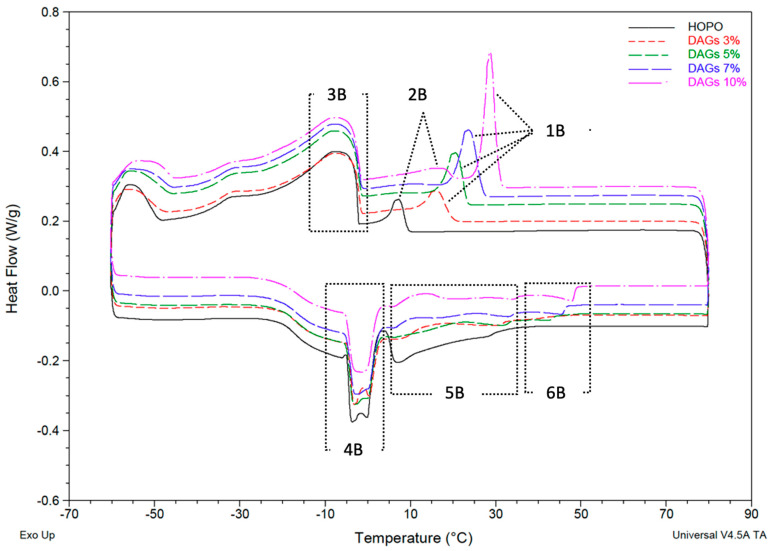
Thermogram for HOPO (black) and DAGs at 3%, 5%, 7% and 10% *n* = 2, with each baseline heat flow offset from HOPO (black) for clarity.

Melted MAG and DAG HOPO oleogels were assessed using DSC, and the resulting curves are shown in Figure 1 and Figure 2. The key crystallization onset/offset temperatures, as well as phase transitions, were identified for each oleogel. The addition of mono- and diglycerides at different concentrations changed the crystallization and melting profiles of the HOPO. With the addition of MAGs, a peak that previously did not exist in HOPO appeared, as observed in Figure 1 (the new peak is at the top right of the cooling curve, labeled “1A”). The contribution of MAGs and DAGs to the early crystallization of the oil can be linked to their behavior when analyzed alone in the DSC. Perez-Santana et al. [29] showed the thermogram of Trancendim 130 with a sharp crystallization peak at 53.4 °C and similarly sharp melting peak with an onset at 30.1 °C and a peak at 49.6 °C. Even though the DSC analysis of Alphadim 90 PBK was not recorded, its thermal behavior can be extrapolated from the composition. Chen et al. [30] reported that this emulsifier is composed of 56.9% palmitic acid and 40.5% stearic acid in the form of monopalmitin and glycerol monostearate, and additionally, the specification of the emulsifier details a melting point of 72 °C, which suggests that crystallization peaks should have an onset around 80 °C. These references allow the deduction that the increase in onset crystallization temperatures is due to the addition of emulsifiers.

There was a positive correlation between onset crystallization temperature and MAG concentration, suggesting the promotion of early nucleation within the system. The appearance of a crystallization peak around 50 °C was also observed by Li and Liu [23] when 4% MAGs were added to corn oil. However, there was a considerable change in peak size from 5% to 7% *w*/*w*. The standard first peak observed in HOPO (labeled in Figure 1 as “2A”) was modified by emulsifier addition, changing the shape, onset temperature and peak size for every increase in the concentration of MAGs. A shift in the onset temperatures was also observed for soybean oil by Si, H. et al. [24], with a peak appearing at 35C with 3% MAG inclusion, and a peak at 42 °C with 6% inclusion. The onset temperature of the peak “3A” for HOPO was not affected by the presence of the monoglyceride (Table 1), as the onset and peak temperatures were in the same position of the thermograms as in the oleogels. This is consistent with the absence of melting peaks below 10 °C for MAGs, specifically glyceryl monostearate [24,27], which suggests that the greatest impact of MAGs as templates for lipid crystal formation is achieved at higher temperatures. For most industrially relevant temperatures, this behavior would be described as the oil fraction suspended in the oleogel.

The presence of MAGs only marginally affected the melting profile of the low melting point fraction by increasing the onset temperature of the first peak (referred to as “4A” in Figure 1) from −5.4 °C in HOPO to −4.7 °C at 10% MAGs (Table 2). The resolution (differentiation of the peaks) of the two events, identified in HOPO as the peak “4A,” increased with the addition of MAGs. This is typical of standard palm oil and related to the melting of β′ crystals followed by α crystals. This suggests a crystalline transition or the coexistence of two polymorphs between −2.8 °C and 0.6 °C (as compiled by Omar et al. [31]). To confirm the transition of structures or the coexistence of two polymorphs, a further study using X-ray diffraction at the same temperature range and heating rate would be necessary.

After the first melting event, the high-melting point fraction of the HOPO (referred to as “5A” and “6A” in the bottom center) broadened in the presence of MAGs; even at the lowest concentrations of MAGs, 3% and 5%, a small peak was evidenced. Similar results have been obtained for olive oil oleogels containing 15% MAGs [32] and are explained as a result of the interaction between the oil and the emulsifier, which creates a less symmetrical packaging upon cooling than that expected for MAGs alone. With the increase in MAGs, there were more intermediate transitions that also increased the melting point. The melting point increased from 30 °C for HOPO to 54.2 °C in the oleogel at 10% MAGs (Table 2). Similar behavior was observed for corn oil + 4% MAG with a last melting peak at 53.8 °C [23] and for melting at 60 °C with 15% MAG [21]. For soybean oil with MAGs at 3% and 6%, the last melting peaks were at 38.3 °C and 46.2 °C, respectively [24].

The presence of an emulsifier that was rich in DAGs (63%) modified the first crystallization event of the HOPO, as seen in Figure 2. Unlike the addition of MAGs, the addition of DAGs between 3 and 7% moved the first crystallization peak (labeled as “1B” from right to left) without creating a new peak. This resulted in peaks at higher temperatures, with larger areas occurring with higher concentrations. The DAGs of 3–7% displaced the initial event observed in the HOPO, unlike the MAGs, which induced an independent crystallization. However, at 10% DAGs, an intermediate melting event “2B” with a peak at 16.2 °C was evidenced (Table 3), suggesting that this emulsifier can indeed act as an early crystallization template, but concentrations above 10% are required.

The previously described melting peak for HOPO “5A” was broadened with the increase in DAGs, suggesting that DAGs regulate polymorphic changes. The presence of DAGs delayed the offset melting point. At 31 °C, the HOPO was completely melted, increasing to 43.1 °C at 3% concentration and 49.3 °C at 10% concentration (Table 4). These results are in accordance with the DSC curves Perez-Santana et al. [29] reported for a high-oleic palm oil shortening containing 5% DAGs prepared with a surface-scraped heat exchanger; the shortening also had a characteristic first melting peak at 24 °C and a second peak at −7 °C.

The naturally occurring diglycerides in HOPO vary in their degrees of saturation. Unsaturated diglycerides 1,3-PalmiticOleic (0.59% *w*/*w*) and 1,3-OleicOleic (0.48% *w*/*w*) are the two most abundant structures [33]. This could explain why the addition of saturated diglycerides modified the existing crystallization curve, as opposed to forming a new peak as was observed with the addition of MAGs, regardless of the initial concentration of DAGs in the HOPO (2.5% *w*/*w*). It has been previously reported that DAGS inhibit nucleation and retard crystal growth of triglycerides, although it seems to be concentration- and DAG-structure-dependent [32]. Similar concentrations to the ones used in this study, 2–10% DAGs, have shown a crystal retardation effect, while concentrations of 30–50% have increased the nucleation rate of palm oil [31]. Conversely, the type of fatty acids contained in the DAGs can dictate the effect of the emulsifier, as an example dipalmitolglycerol has shown a fast crystallization in palm oil while palmitoylglycerol seemed to retard it [34].

### 2.2. Textural Analysis

Based on the DSC results and common operation temperatures in industrial settings, texture analyses were performed at 15 °C, 10 °C and 5 °C. The oils were cooled at the same rate and conditioned at test temperatures for 24 h to promote uniform gel structure formation. The hardness of both types of oleogels increased with the emulsifier concentration. A linear increase in the hardness of oleogels with MAG concentrations has been confirmed by several authors [21,28], explained by the presence of more crystal formations supporting the network.

**Figure 3:**
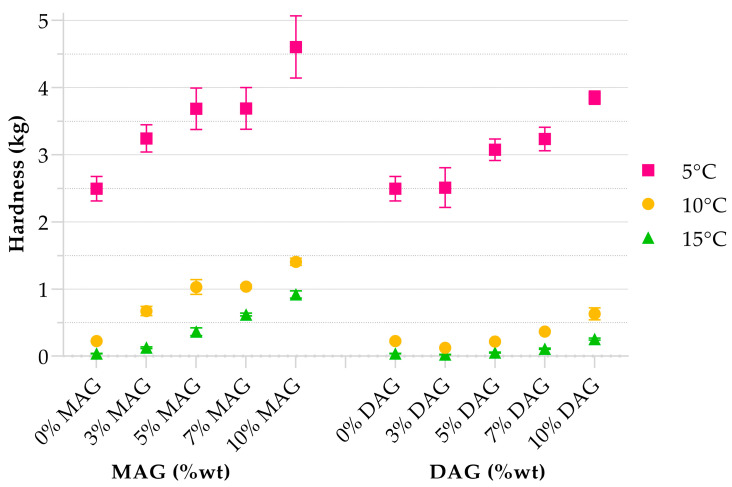
Hardness of MAG and DAG oleogels at 0%, 3%, 5%, 7% and 10% of respective emulsifiers at 5 °C, 10 °C and 15 °C. *n* = 4 for all measurements.

Hardness values increased at lower temperatures (Figure 3). The hardest HOPO oleogel was achieved using MAGs at a concentration of 10% at 5 °C (Table 5). All oleogels were harder than HOPO at all temperatures, implying that the emulsifier did change the dispersion of solid fats within the liquid oil, strengthening the structure. However, there were no significant differences between the hardness values of MAGs between 5% and 7% at any temperature (Table 5). The effect of temperature on the hardness of oleogels does not seem to be linear but rather dependent on the amount of crystallized fat achieved by the MAG effect in the context of different oil sources. Previous works [24] similarly reported no/little effect on texture from the addition of 3% MAG to soybean oil between 5 and 25 °C, while a major differentiation was observed as MAG concentrations increased.

The hardest DAG-HOPO oleogel was identified at 10% emulsifier and 5 °C for the crystallization temperature, with a hardness of 3.8 kg. There was not a significant difference in hardness at 5 °C between 0% and 3% and between 5% and 7% emulsifier (Table 5). For all HOPO oleogels, there was a considerable increase in hardness between 10 °C and 5 °C, suggesting a higher concentration of solid fat at lower temperatures. When compared with the thermogram in Figure 2, all first crystallization events had already fully developed at 10 °C; therefore, there should be a major crystal aggregation below 10 °C. While DAGs were effective at modifying the thermophysical properties of HOPO in a similar manner to MAGs, the effect was muted when compared at equal concentrations and temperatures.

A previous study by Perez-Santana et al. [29] reported on 5% DAGs HOPO shortening with a hardness of 0.01 kg at 21 °C, which agrees with the trend of 5% DAGs oleogel in this study with 0.057 kg at 15 °C, supporting the correlation of increasing temperatures resulting in lower hardness of this oleogel. However, in the same study, the lower hardness of the shortening did not cause a lower hardness of cookies; instead, the saturated fatty acid composition seemed to govern the cookie texture. The possibility to use a soft oleogel and obtain a similar performance to a commercial solid baking fat suggests a lower complexity in fat transportation and processability, which could result in a lower cost of goods.

The work of penetration showed a similar trend to the hardness of the oleogels, mainly increasing with temperature, although emulsifier concentration was also a statistically significant variable. MAG oleogels required more energy for penetration, compared to DAG oleogels, with significant differences at 10 °C and 15 °C, while at 5 °C and concentrations of 3%, 5% and 10% emulsifier, both oleogels exhibited similar resistance to penetration (similar areas under the curve), see Table 6; this could be due to differences in their macrostructures, an oleogel with DAG that results in a dense and homogeneously structured matrix may have a fast increase in the initial energy for penetration and a plateau at the force required to penetrate. In the case of MAGs, there may be a gradient from harder to softer portions of oleogel from the core to the edges, which causes an incremental amount of force to penetrate, causing higher peak forces but similar areas under the curve compared to DAGs.

### 2.3. Polarized Light Microscopy (PLM)

**Figure 4:**
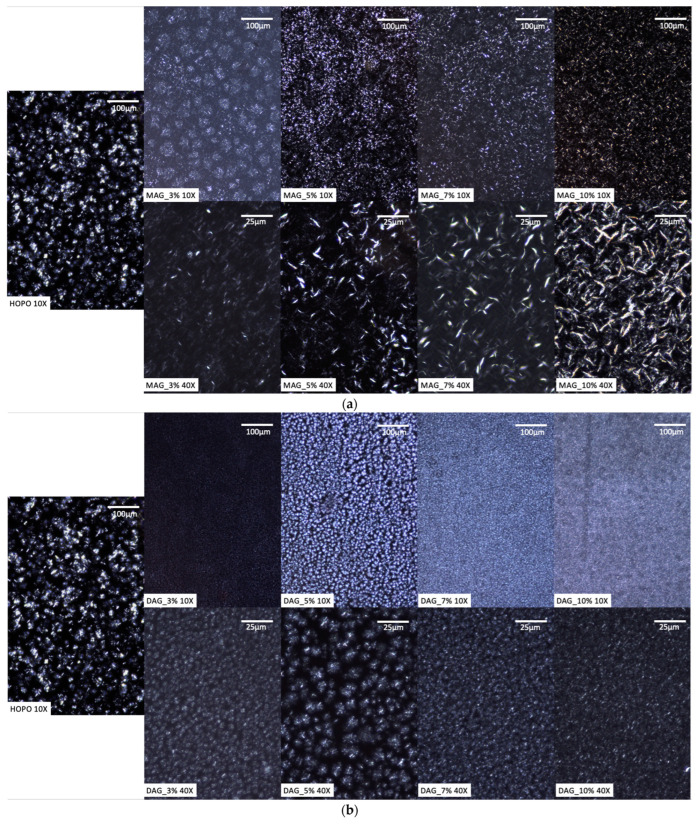
(**a**) Polarized light microscopy of oleogel crystals created with increasing concentrations of MAGs at 10 °C. (**b**) Polarized light microscopy of oleogel crystals created with increasing concentrations of DAGs at 10 °C.

Figure 4a,b shows the microstructure of fat crystals at increasing levels of emulsifier addition. In Figure 4a, there was an increase in the size and abundance of needle-like shapes with higher concentrations of MAGs. Perța-Crișan et al. [35] observed that MAGs had a similar influence on fat crystal shapes (mainly forming needle-like crystals) when used as oleogelators in other oils, including olive oil, sunflower oil and high-oleic sunflower. Other structures, such as spherulites or rosette-like structures, have been observed when used with corn oil [36], suggesting that there are likely additional factors which influence crystal shape. Regardless, Figure 4a highlights the capacity of monoglycerides to form a strong laminar crystal network, which translated into early crystallization peaks and higher mechanical strength for HOPO oleogels at concentrations of as low as 3% MAGs. Kupiec et al. [37] also reported harder oleogels with monoglycerides, which produced a densely packed and evenly distributed organogel network, and the growth of this crystal network over time was also recorded as the reason why the hardness of oleogels increases upon storage [38].

Conversely, Figure 4b illustrates the effect of DAGs on oleogel crystallization, which resulted in spherulite-shaped crystal formation over needle-shaped. It should be noted that the crystals (while more numerous) were similar in size to those found in pure oil at lower concentrations (3–5%), while at higher concentrations (7–10%), smaller spherulites with a higher abundance were observed. These effects of DAGs have also been observed for coconut oil and palm kernel oil oleogels [39] and have been explained by a faster formation of crystalline nuclei when DAGs are included, resulting in finer and more uniform crystals. While these changes did affect the thermomechanical properties of the DAG-based oleogels, the effects were not as significant as with MAGs at equal concentrations.

## 3. Conclusions

The use of mono- and diglycerides changed the thermal and mechanical properties of HOPO (a novel oil with health-promoting micronutrients and a lower saturated fatty acid content compared to standard palm oil), thereby broadening its potential industrial applications. The addition of commercially available saturated monoglycerides promoted an early nucleation stage that was not previously present in HOPO, and microscope images showed needle-like networks increasingly growing with higher concentrations of MAGs. The hardness of the oleogel generally increased with the addition of emulsifier. The hardest oleogel (4.6 kg) was obtained by using 10% *w*/*w* monoglyceride at a crystallization temperature of 5 °C; however, at these lower temperatures, MAG and DAG oleogels required similar energy levels for deformation, suggesting a more homogeneous structure in DAGs and a gradient of gel strength from the edges of the vials to the core with MAGs.

The commercially available saturated diglyceride DAGs also had major effects on the thermal and mechanical characteristics of HOPO by dominating the initial crystallization stage of the oleogel, as was also observed under microscopy as a promoter of small spherulite fat crystals. The onset crystallization temperature also increased with the addition of DAGs, promoting the early crystallization of the high-melting fraction of the HOPO. DAGs had less of an effect than MAGs on the hardness of the oleogel. The maximum hardness (3.8 kg) for DAG oleogels was also obtained at the highest (10% *w*/*w*) diglyceride concentration at the lowest assessed temperature (5 °C).

The addition of commercially available mono- and diglyceride-based emulsifiers is an option to increase the range of applications of HOPO by incorporating the liquid and solid phases of the oil, creating stable oleogels of industrial interest. The addition of MAGs and DAGs in various concentrations, as seen in this study, allows companies to personalize their formulations to achieve desired results that may not have been possible previously, thereby facilitating creativity and profitability within the industry.

## 4. Materials and Methods

### 4.1. Materials

**Figure 5:**
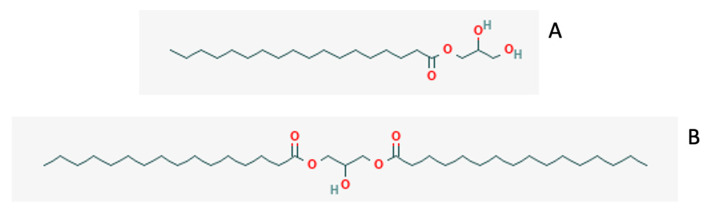
General structure of emulsifiers used in this study. (**A**) MAGs mainly containing stearic and palmitic fatty acids. (**B**) DAGs mainly containing palmitic and oleic fatty acids.

The crude oil of the high-oleic palm oil (HOPO) was obtained using mechanical press extraction from the interspecific hybrid *E. guineensis* and *E. oleifera* OxG. The oil was then refined, bleached and deodorized. Specifications of the oil included 0.1% maximum acidity (as oleic acid), 0.05% maximum moisture, minimum iodine value (IV) of 67, maximum peroxide value (PV) of 1 and a maximum melting point of 27 °C. It had an SFC of 5–13% at 15 °C and 2–5% at 20 °C. This oil was provided by Thin Oil Products (Weston, FL, USA). The fatty acid composition of the HOPO used in this study is provided in Table 7. Commercially available emulsifiers Alphadim 90 PBK and Trancendim 130 were donated by Corbion Ingredients (Lenexa, KS, USA). Alphadim 90 PBK (referred to as MAG) is 95% monoglycerides, and Trancendim 130 (referred to as DAG) is 67% diglycerides, 27% triglycerides and 6% monoglycerides. The structures of these emulsifiers are shown in Figure 5.

FA composition analysis was performed by FoodChain ID Testing Laboratories. HOPO is High-oleic palm oil. “Other FAs” include saturated, mono- and poly- unsaturated fatty acids which were not detected by the AOCS Ce-1b-89 method as reported by FoodChain ID Testing Laboratories.

### 4.2. Oleogel Preparation

**Figure 6:**
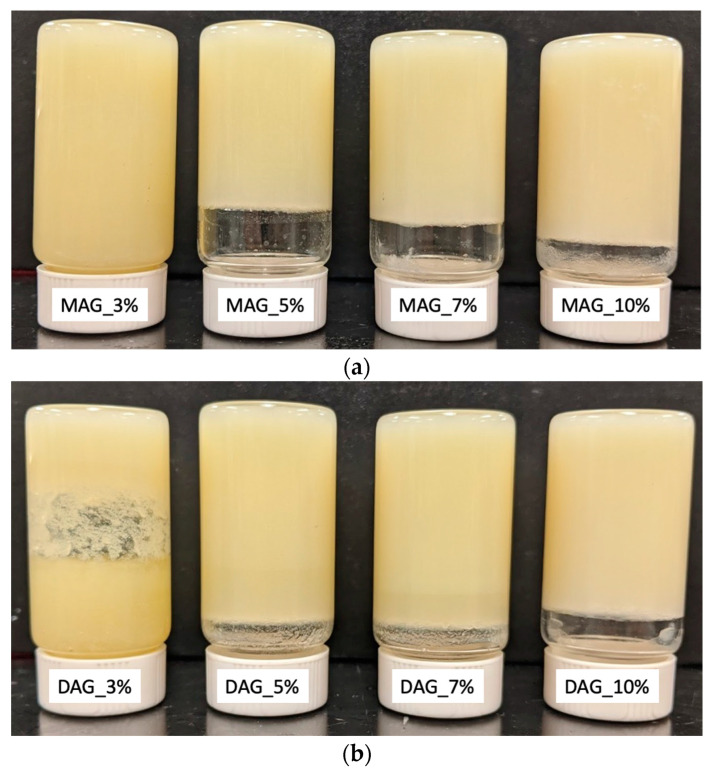
Inverted tubes of prepared HOPO oleogels with (**a**) 3%, 5%, 7% and 10% MAG and (**b**) 3%, 5%, 7% and 10% DAG, cooled down to 20 °C.

HOPO was mixed with Alphadim 90 PBK or Trancendim 130 to form 3%, 5%, 7% and 10% *w*/*w* dispersions of each emulsifier, for a total of 8 different samples: MAG_3%, MAG_5%, MAG_7%, MAG_10%, DAG_3%, DAG_5%, DAG_7% and DAG_10%. First, 1 L glass beakers of HOPO were immersed in a water bath and heated at 80 °C to melt all solid fatty acids and erase crystal memory. Aliquots of HOPO were portioned into 100 mL glass beakers. Each emulsifier was added in its corresponding percentage at 75 °C until the dispersion was clear; it was further stirred by hand with spatula every 2 min for a total of 10 min, and samples were allowed to heat up to 80 °C. The cooling of samples was critical for textural analysis, as described below. Aliquots of 15 g for each sample were transferred into 20 mL screw-capped glass vials (*n* = 4) and then transferred to sequential water baths at 45 °C, 40 °C, 35 °C and 30 °C to control the cooling transition temperature at ΔT = 25 °C. Upon reaching equilibrium, each sample was transferred to a second water bath at 15 °C, 10 °C and 5 °C, respectively, and conditioned for 24 h. This procedure was intended to create fat samples under identical cooling and shear conditions (0 s^−1^), carefully controlling the crystallization by temperature changes until equilibrium was reached. Before conducting thermomechanical tests, the confirmation of the formation of oleogels was achieved through the use of the inverted test tube method [40]. From each of the water baths (15 °C, 10 °C and 5 °C), a distinct group of vials was extracted and placed upside down in their caps. They were considered fully gelled when the material did not exhibit gravitational flow after 5 min. Figure 6 shows the results of this test for 20 °C, where concentrations of both DAGs and MAGs at 5% or greater resulted in gel formation. At temperatures of 5, 10 and 15 °C, all concentrations resulted in gel formation.

### 4.3. Differential Scanning Calorimetry

A differential scanning calorimeter (DSC) Q2000 (TA Instruments) was used to assess the thermomechanical properties of both oils. Melted oleogel samples of between 6.0 and 9.0 mg were weighed into aluminum pans and hermetically sealed at 75 °C. An empty hermetically sealed aluminum pan was used as a baseline. All the samples followed the AOCS Cj 1–94, 2009 method described by Maruyama et al. [41]—an 80 °C isotherm for 10 min, cooldown at a rate of 10 °C/min to −60 °C and heating at a rate of 10 °C/min to 80 °C. All the samples underwent the same heating/cooling program and were run in duplicate. Onset and offset temperatures were determined for each identified peak as the intersection point between the extrapolated baseline and the tangent line drawn at the inflection point of the peak. TA Universal Analysis software was used for the identification of the onset, offset, peak temperatures and for illustration of the results.

### 4.4. Textural Analysis

Samples were removed from their respective water baths at 15 °C, 10 °C and 5 °C (the same as described in Section 2.1) one glass vial at a time and immediately analyzed to examine the effect of temperature in the texture of the different oleogels. The test was performed immediately after removing the samples from the water baths; this ensured negligible temperature changes up to the execution of the tests, as each sample was analyzed within 1 min from removal of the water bath. The puncture test was performed using a texture analyzer TA-XT Plus (Stable Micro Systems, Scarsdale, NY, USA) equipped with a 6 mm diameter cylindrical probe at room temperature. The pretest speed was 1 mm/s, test and post-test speeds were 2 mm/s and distance of penetration was 12 mm. Hardness (maximum positive force) and work of penetration (area under the curve) were measured when the probe compressed the sample. The test was performed in four replications. Two-way ANOVA at α = 0.05 followed by a Tukey HSD test were used to identify differences between treatments (crystallization temperature and emulsifier concentration). The mean separation was used to denote significant differences.

### 4.5. Statistical Analysis

Two-way ANOVA was used to identify differences between means for the textural analysis, and Tukey’s HSD test was used to determine the statistical significance. The level of significance for all tests was α = 0.05. Statistical analyses were performed with GraphPad Prism 8.0 (GraphPad Software, Inc., San Diego, CA, USA).

### 4.6. Polarized Light Microscopy (PLM)

The microstructures of oleogels with MAGs and DAGs were captured at 10 °C to illustrate the differences in crystal shapes and abundance between both emulsifiers. These were imaged using PLM (T610D microscope, Irvine, CA, USA). A total of 10 μL of each melted oleogel sample was dropped on a clean glass slide, and a coverslip was carefully placed on the top to ensure uniform thickness. A controlled temperature platform was adapted to the microscope, with inner channels for coolant fluid flowing in and out from a recirculation water bath. The slides went through the same stepwise temperature cooling ramp as described in Section 2.3. PLM images were captured at 10× and 40× magnification after the slides reached 10 °C for 24 h.

## Figures and Tables

**Table 1:** Crystallization temperatures for MAG oleogels at 0%, 3%, 5%, 7% and 10% (*w*/*w*).

	Temperatures (°C)					
MAGs (%)	T_onset_ 1A	T_peak_ 1A	T_onset_ 2A	T_peak_ 2A	T_onset_ 3A	T_peak_ 3A
0			10 ± 0.8	7.4 ± 0.6	−2.1 ± 0.1	−7.9 ± 0.7
3	31.3 ± 0.6	29.8 ± 0.4	11.8 ± 0.2	7.9 ± 0.7	−1.9 ± 0.2	−7.2 ± 0.2
5	39.3 ± 0.7	37.2 ± 1.5	11.8 ± 0	9.5 ± 0.3	−1.8 ± 0.2	−7.5 ± 0.5
7	41.2 ± 0	39.6 ± 0.8	11.8 ± 0.4	9.9 ± 0.2	−1.9 ± 0.2	−7.6 ± 0.2
10	45.3 ± 0.1	44 ± 0.3	11.6 ± 0.1	10.2 ± 0.1	−2 ± 0.3	−7.5 ± 0.6

**Table 2:** Melting temperatures for MAG oleogels at 0%, 3%, 5%, 7% and 10% (*w*/*w*).

	Temperatures (°C)								
MAGs (%)	T_onset_ 4A	T_peak_ 4A,1	T_peak_ 4A,2	T_offset_ 4A	T_onset_ 5A	T_peak_ 5A	T_offset_ 5A	T_peak_ 6A	T_melt_
0	−5.4 ± 0	−3.9 ± 0.2	−0.4 ± 0.1	3.4 ± 0	3.4 ± 0	6.9 ± 0.6			30.8 ± 1
3	−4.5 ± 0.1	−2.5 ± 0.4	1.2 ± 0.5	4.6 ± 2.3	10.7 ± 1.9	15 ± 2.1	29.4 ± 0.3	41.1 ± 0.6	46.1 ± 3.2
5	−4.7 ± 0.1	−2.5 ± 0.7	1.1 ± 0.3	4 ± 1.6	10.7 ± 0.8	14.4 ± 2.7	30 ± 1.3	47.2 ± 0.2	51.4 ± 1.7
7	−4.7 ± 0	−2.4 ± 0.4	1.1 ± 0.1	4.1 ± 1	10.7 ± 0.5	12.2 ± 0.1	29.4 ± 0.3	49.4 ± 0.2	51.9 ± 1.6
10	−4.7 ± 0	−2.4 ± 0.8	1.2 ± 0.5	4.5 ± 1.7	11.1 ± 0.8	12.3 ± 0.9	29.9 ± 1.6	52.4 ± 0.3	54.2 ± 1.2

**Table 3:** Crystallization temperatures for DAG oleogels at 0%, 3%, 5%, 7% and 10% (*w*/*w*).

			Temperatures (°C)			
DAGs (%)	T_onset_ 1B	T_peak_ 1B	T_onset_ 2B	T_peak_ 2B	T_onset_ 3B	T_peak_ 3B
0			10 ± 0.8	7.4 ± 0.6	−2.1 ± 0.1	−7.9 ± 0.7
3	19.2 ± 0.6	16.1 ± 0.1			−1.7 ± 0.3	−7.7 ± 0.7
5	23.1 ± 0.7	20.6 ± 1			−1.6 ± 0.1	−8 ± 0.7
7	27 ± 0.1	24 ± 0.5			−1.7 ± 0.4	−7.8 ± 0.5
10	30.5 ± 0.3	28.5 ± 0.6	20.1 ± 0.2	16.2 ± 0.3	−1.6 ± 0.2	−7 ± 0.8

**Table 4:** Melting temperatures for DAG oleogels at 0%, 3%, 5%, 7% and 10% (*w*/*w*).

	Temperatures (°C)							
DAGs (%)	T_onset_ 4B	T_peak_ 4B,1	T_peak_ 4B,2	T_offset_ 4B	T_onset_ 5B	T_peak_ 5B	T_peak_ 6B	T_melt_
0	−5.4 ± 0	−3.9 ± 0.2	−0.4 ± 0.1	3.4 ± 0	3.4 ± 0	6.9 ± 0.6		30.8 ± 1
3	−4.9 ± 0.3	−2.8 ± 0.6	0.8 ± 0.2	4.2 ± 2.3	22.2 ± 2.6	29.7 ± 0.7	40.1 ± 0.9	43.1 ± 1.6
5	−5.3 ± 0.1	−2.6 ± 0.8	0.6 ± 0.4	5.7 ± 4.2	25.4 ± 0.7	31.4 ± 0.6	43 ± 0.5	44.5 ± 0.7
7	−5.4 ± 0.1	−2.5 ± 0.4	0.6 ± 0.1	5.5 ± 4	27.7 ± 1.4	32.6 ± 0.6	45.7 ± 0.3	47.2 ± 0.9
10	−5.7 ± 0	−1.3 ± 1.4	0 ± 0	3.2 ± 1.4	29.9 ± 1.2	33.4 ± 1	47.9 ± 0	49.3 ± 0.7

**Table 5:** Hardness of MAG and DAG oleogels at different concentrations and final cooling temperatures.

		Hardness (kg)							
T (°C)	HOPO	MAG 3%	MAG 5%	MAG 7%	MAG 10%	DAG 3%	DAG 5%	DAG 7%	DAG 10%
5	2.49 ± 0.18 ^d^	3.24 ± 0.20 ^c^	3.69 ± 0.31 ^b^	3.69 ± 0.30 ^b^	4.61 ± 0.46 ^a^	2.51 ± 0.30 ^d^	3.07 ± 0.16 ^c^	3.24 ± 0.17 ^c^	3.85 ± 0.10 ^b^
10	0.23 ± 0.02 ^d^	0.67 ± 0.07 ^c^	1.03 ± 0.12 ^b^	1.04 ± 0.03 ^b^	1.41 ± 0.05 ^a^	0.12 ± 0.01 ^d^	0.22 ± 0.02 ^d^	0.37 ± 0.02 ^cd^	0.63 ± 0.09 ^c^
15	0.04 ± 0.00 ^c^	0.13 ± 0.01 ^c^	0.37 ± 0.06 ^bc^	0.62 ± 0.02 ^ab^	0.92 ± 0.05 ^a^	0.02 ± 0.0 ^c^	0.07 ± 0.01 ^c^	0.11 ± 0.01 ^c^	0.26 ± 0.02 ^bc^

T: Temperature. Different letters represent significant differences within the same row (temperature) at *p* < 0.05 probability level (Tukey’s test).

**Table 6:** Work of penetration (WP) for MAG and DAG oleogels at different concentrations and final cooling temperatures.

		WP (kg.s)							
T (°C)	HOPO	MAG 3%	MAG 5%	MAG 7%	MAG 10%	DAG 3%	DAG 5%	DAG 7%	DAG 10%
5	6053.8 ± 594.8 ^d^	7871 ± 577.8 ^c^	10,100 ± 828.5 ^ab^	10,775 ± 278.9 ^a^	10,879 ± 1595 ^a^	7388 ± 448.6 ^c^	9552 ± 501.6 ^b^	9104 ± 887.1 ^b^	10,764 ± 1266 ^a^
10	700.7 ± 33.6 ^d^	2191 ± 165.5 ^bc^	3167 ± 342.5 ^b^	3254 ± 95.8 ^b^	4507 ± 130.4 ^a^	388.2 ± 13.6 ^d^	672.7 ± 75.6 ^d^	1136 ± 67.4 ^cd^	2054 ± 225.6 ^c^
15	115.2 ± 8.5 ^c^	418.9 ± 47.5 ^c^	1120 ± 180.7 ^bc^	1956 ± 54.6 ^ab^	2849 ± 173.0 ^a^	62.9 ± 10.6 ^c^	154.7 ± 29.2 ^c^	338.6 ± 27.3 ^c^	816.2 ± 32.5 ^bc^

T: Temperature. Different letters represent significant differences within the same row (temperature) at *p* < 0.05 probability level (Tukey’s test).

**Table 7:** Fatty acid composition (% *w*/*w*) of HOPO used in this study.

Fatty Acids (FA)	HOPO (% *w*/*w*)
C8:0	0.01
C10:0	0.02
C12:0	0.2
C14:0	0.4
C15:0	0.1
C16:0	27.8
C17:0	0.1
C18:0	2.8
C20:0	0.3
C22:0	0.1
C16:1	0.3
C18:1	55.5
C20:1	0.1
C18:2	11.9
C18:3 (𝛾)	0.2
Saturated FA	31.8
Monounsaturated FA	56
Polyunsaturated FA	12.2
Other FAs	0.1

## Data Availability

Data presented within the article.
